# The EndoBarrier: Duodenal-Jejunal Bypass Liner for Diabetes and Weight Loss

**DOI:** 10.1155/2018/7823182

**Published:** 2018-07-26

**Authors:** Aruchuna Ruban, Hutan Ashrafian, Julian P. Teare

**Affiliations:** Department of Surgery and Cancer, Imperial College, London, UK

## Abstract

The rapid rise of obesity and type 2 diabetes poses a global threat to healthcare and is a major cause of mortality and morbidity. Bariatric surgery has revolutionised the treatment of both these conditions but is invasive and associated with an increased risk of complications. The EndoBarrier is a device placed endoscopically in the duodenum, which is designed to mimic the effects of gastric bypass surgery with the aim of inducing weight loss and improving glycaemic control. This review outlines the current clinical evidence of the device, its efficacy, potential mechanisms of action, and utility in clinical practice.

## 1. Introduction

Obesity has reached epidemic proportions with the WHO estimating that approximately 2.3 billion adults worldwide are overweight and more than 700 million are obese [1]. Obesity is associated with the development of other comorbidities, in particular diabetes, which currently affects 422 million adults worldwide [2]. The current favoured treatment for patients with type 2 diabetes (T2DM) who are obese is referral for bariatric surgery. In the 2nd Diabetes Surgery Summit in 2015, several national diabetes societies such as the American Diabetes Association (ADA) and Diabetes UK have recommended the use of bariatric surgery in obese type 2 diabetics reporting diabetes remission rates of between 30–60% following surgery [3]. However, with a greater demand being placed on bariatric surgery, there is a drive to develop nonsurgical alternatives to combat the ever-rising obesity and diabetes epidemic.

One potential alternative is the EndoBarrier, an endoluminal duodenal-jejunal bypass liner (DJBL) developed by GI Dynamics (GID) Inc., Lexington, MA. In this state-of-the-art review, we present the current clinical trial data available on the EndoBarrier and explore its potential mode of action, safety profile, and potential applications in the management of obesity and T2DM.

## 2. The Device

**Figure 1:**
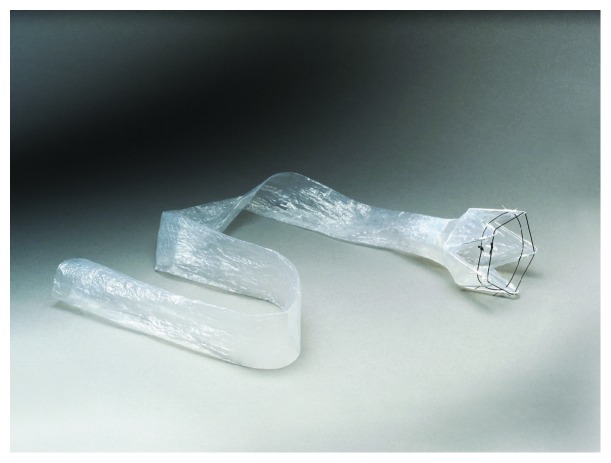
The EndoBarrier device.

**Figure 2:**
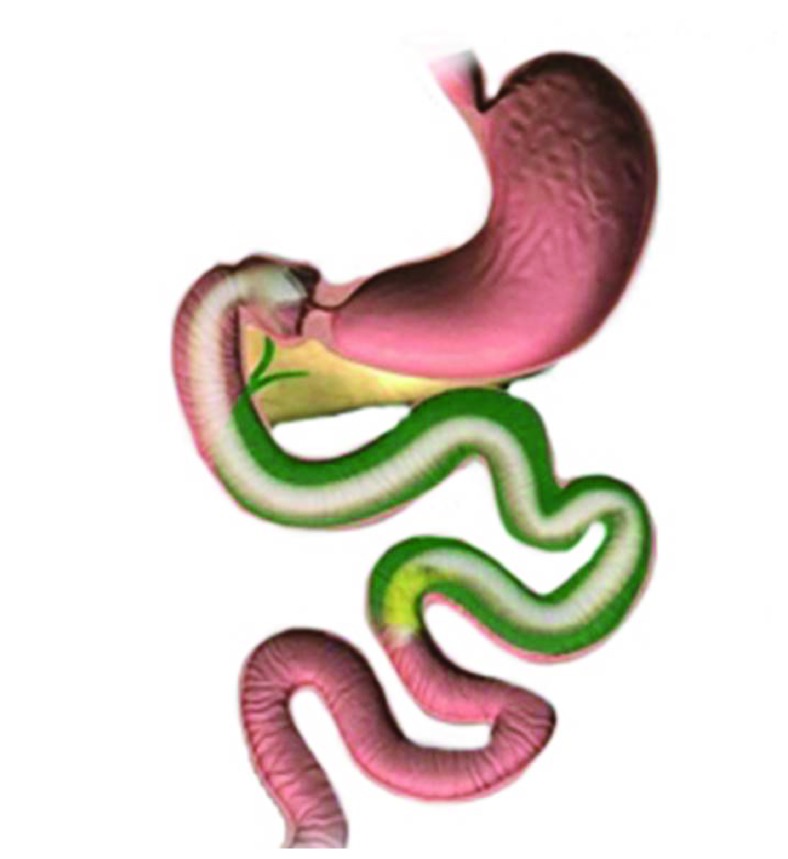
Bile flowing around the outside of the sleeve coming into contact with nutrients distally in the jejunum.

The EndoBarrier is a single-use endoscopic implant, which consists of the liner, delivery system, and retrieval system. The actual liner portion is an impermeable fluoropolymer that spans 60 cm into the small intestine (Figure 1). Located at the proximal end of the liner are anchors with barbs made of nitinol allowing the device to affix and be secured to the duodenal bulb, proximal to the ampulla of Vater but distal to the pylorus. These anchors also have drawstrings attached to them to facilitate subsequent removal of the device at the end of treatment. Once deployed in the duodenum, the device can remain for a maximum treatment period of 12 months. The liner is open at both ends promoting the passage of chyme from the stomach bypassing the duodenum and into the jejunum. Concurrently, pancreatic juices and bile will enter the duodenum from the ampulla of Vater running along the outside of the sleeve avoiding contact with gastric contents until these exit the sleeve in the jejunum (Figure 2). The desired effect is for the sleeve to mimic the duodenal-jejunal exclusion portion of gastric bypass surgery, thus recreating the beneficial effects seen in postsurgery on glucose homeostasis and energy metabolism, but without enduring permanent alterations to the intestinal anatomy and avoiding the risks associated with undergoing invasive surgery.

### 2.1. Procedural Overview

All patients undergoing EndoBarrier implantation will adhere to strict dietary advice, which involves following a liquid diet of nutritional drink supplements for at least 1 week prior to their implant date and for 1-2 weeks of postprocedure. The purpose of following this regime is to facilitate clear views during endoscopy for implantation of the device and to minimise the risk of a food bolus obstruction of the liner in the first few weeks of postprocedure.

Both general and conscious sedation can be used for this procedure, which also requires fluoroscopic imaging for guidance in the insertion or removal of the liner. A thorough endoscopic examination of the upper GI tract is conducted prior to device placement to ensure suitability and to detect any potential anatomical variants such as a short duodenal bulb or peptic ulcer disease, which may preclude the device from being inserted. GID, the manufacturer of the device, advocates prescribing a proton pump inhibitor (e.g., omeprazole 40 mg twice daily) three days prior to insertion and to continue this whilst the device is in situ up until two weeks after the device is removed to minimise the risk of potential bleeding from the implant. Furthermore, to reduce the potential for infection, GID also suggests administering a single dose of broad-spectrum antibiotic (e.g., 2 g ceftriaxone) 1-2 hours prior to implantation.

The advantages and disadvantages of the EndoBarrier implant and explant procedure are summarised in Table 1.

### 2.2. Implantation

**Figure 3:**
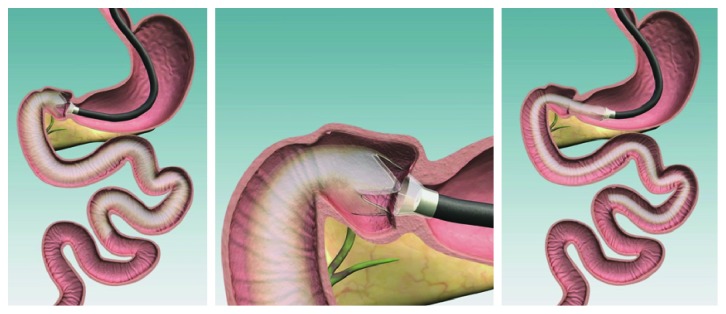
Duodenal sleeve implantation.

Implantation takes on average 45 minutes to perform. The patient is placed in left lateral decubitus position, and a surveillance gastroscopy is performed after which the endoscope is placed in the first part of the duodenum. A guidewire is advanced as distally as possible into the duodenum through the working channel of the gastroscope and the gastroscope is removed. The EndoBarrier delivery system (coaxial catheter system with capsule containing the EndoBarrier sleeve) is advanced over the guidewire until the capsule sits comfortably in the pylorus. This is aided by a dotted line marked on the capsule, which allows the correct positioning of the device in the pylorus, so the distal end sits in the duodenal bulb ready to deploy the sleeve. The guidewire is then removed. The delivery system has 5 steps, which are followed in order to release the device from its protective capsule into the ideal position, which should be 5–10 mm from the pylorus with the proximal barbs anchored in the duodenal bulb and the sleeve running distal to this (Figure 3). Peristalsis will ensure the sleeve unravels across its 60 cm length into the duodenum. A water-soluble contrast such as gastrografin is then used to confirm the positioning of the device and to prove it is patent. The patient is usually discharged at home the same day following recovery from the anaesthetic.

### 2.3. Explantation

Explantation takes on average 30 minutes to perform. The patient is placed in left lateral decubitus position, and a surveillance gastroscopy is performed. The endoscope is then removed, and a protective hood is placed at the end of the endoscope. This circular hood is made out of a durable plastic polymer and is designed to hold within it the sharp anchors located at the proximal end of the liner. The endoscope is reintroduced into the duodenum, and the anchors of the device are identified. Each of the anchors has drawstrings attached to them. A specially designed grasper with a hook at the end contained in a protective plastic sheath is then passed down to the therapeutic channel of the scope until it arrives distal to the protective hood at the end of the endoscope. The retrieval hook is then advanced forward beyond its sheath and positioned around one of the drawstrings before being retracted, pulling the drawstring with it, thus collapsing down the device. Once the anchor is fully collapsed, the retrieval device is locked in position, and the endoscope with hood is advanced forward ensuring that all the collapsed anchors are captured within the hood, which can be confirmed by fluoroscopy. Under fluoroscopic guidance, the endoscope retrieval system and liner are removed together, making sure they can travel safely through the upper GI anatomy. Finally, the explant site is examined for signs of bleeding. The patient is usually discharged at home the same day following recovery from the anaesthetic.

## 3. Clinical Trial Data

To date, there have been five randomised controlled trials (RCTs) examining the efficacy of the EndoBarrier (Table 2). The largest of which was a multicentered trial performed in the Netherlands, in which 73 patients were randomised to receive either DJBL treatment in combination with dietary intervention or dietary intervention alone (the control group) [4].

A total of 35 patients successfully had the EndoBarrier implanted for a 6-month period. BMI at baseline was 35 kg/m^2^ and 37 kg/m^2^ in the device arm and control arm, respectively, and reduced to 31 kg/m^2^ and 35 kg/m^2^, respectively, over the 6-month period. HBA1c at baseline was 8.3% in both groups and reduced to 7.0% and 7.9% in the device and control arm, respectively. There was only one early device removal due to blockage of the DJBL with food. Patients were also followed up postremoval of the device after 6 months where BMI and HBA1c were measured. BMI was 32 kg/m^2^ in the device group and 36 kg/m^2^ in the control group, respectively, showing a slight increase following the device removal in the treatment arm. HBA1c was 7.3% and 8.0% at the end of the follow-up period in both groups, showing a mean reduction of 1% and 0.3% in both groups, respectively.

In another study of 41 patients, 26 had the device implanted compared to a control group on a low-calorie diet and had a mean excess weight loss of 19% for the device versus 6.9% in the control group [5]. Furthermore, out of 8 patients in the device arm with T2DM at baseline, improvements were seen in glucose levels and HBA1c in all but one of them.

Our research group at Imperial College conducted the first postmarketing clinical trial of the EndoBarrier in the UK consisting of 45 patients recruited from three centers (St. Mary's Hospital London, Southampton, and University Hospital Manchester) [9]. In this study, participants were aged 18–65 years with T2DM, with a BMI greater than 30 kg/m^2^ and received the implant for a duration of one year. Mean HBA1c and BMI at baseline were 69 mmol (8.5%) and 39.9 kg/m^2^, respectively. Of the 45 patients, 31 (69%) completed the 12-month study period. Average implantation time was 27 minutes, and fluoroscopic time was 7 min (SD 5.7). There were no procedure-related complications during implant or explant. There were 14 early withdrawals before the 12-month implant period, and two of these participants had device-related adverse events requiring premature explant for melaena and device migration resulting in abdominal pain, respectively. The other reasons for withdrawal included the development of other medical complications precluding EndoBarrier implantation and patient choice for early removal.

At 1 year at the time of explant, the average reduction in HBA1c was 0.8%. A mean reduction in BMI of 4.9 kg/m^2^ was observed with a mean weight loss of 15 kg. These positive changes appeared to be maintained at the 6-month follow-up period with small but insignificant changes in these parameters after explantation.

A recent systematic review and meta-analysis assessing the effect of the EndoBarrier on glycaemic control in obese patients with T2DM yielded similar results [10]. This included 388 patients from the 5 RCTs listed above and additional 9 observational studies with an average device implantation time of 8.4 ± 4 months. HBA1c decreased by 1.3% at the time of explantation. The effect of the EndoBarrier on weight loss was also investigated, which included 10 studies with a total of 352 patients. Average weight loss was 11.3 kg at time of explantation corresponding to a BMI reduction of 4.1 kg/m^2^. The average device implantation in this cohort of patients was 9.2 ± 3.1 months.

## 4. Potential Mechanisms of Action

There is currently a poor understanding of mechanisms underpinning how the device elicits its effects on weight loss and glycaemia. As the EndoBarrier is designed to mimic the bypass portion of the RYGB surgery, one might postulate that it works by similar mechanisms. One potential theory of how RYGB surgery works is the so-called BRAVE effects (bile flow changes, restriction of stomach size, anatomical gastrointestinal rearrangement, vagal manipulation, and enteric hormonal modulation) [11]. These changes are believed to take place within minutes of the RYGB surgery being performed and are considered as triggering gears that activate a multitude of subsequent downstream mechanisms (hindgut, foregut, and midgut theories), which might account for why glycaemic improvements are observed independent of any weight loss occurring. The EndoBarrier was designed to be an “endoscopic bypass” so that it has been envisaged to activate some of the same mechanisms as bariatric operations (historically based on foregut exclusion or the “foregut theory” where a putative anti-incretin is no longer released after surgical/interventional foregut exclusion). We consider some of the likely mechanisms including

- enteric hormonal modulation and the incretin theory,
- alterations in the gut microbiota,
- bile flow changes.

As very little data currently exists in the literature on how the EndoBarrier influences these above mechanisms, most of the evidence described below will be drawn from our experience post-RYGB surgery.

### 4.1. Incretin Theory

It is now widely accepted that altering the gastrointestinal anatomy following RYGB alters the flow of nutrients leading to important changes in gut-derived hormones by foregut exclusion and modified hindgut signals. This in turn positively influences the metabolic changes seen following surgery including improvement in glycaemic control and weight loss. Fundamental to this is the paradigm of the “incretin effect,” which is the concept that insulin secretion is also directly influenced by hormonal cues resultant from food intake and energy expenditure [12]. In fact, it is estimated that the incretin effect accounts for as much as 50–70% of insulin secretion in response to an oral glucose load [13]. However, this incretin response is thought to be significantly impaired in patients who are obese or have T2DM [14]. Two key incretin hormones are glucagon-like peptide-1 (GLP-1) and glucagon-dependent insulinotropic polypeptide (GIP), which are both released following a meal from intestinal enteroendocrine cells known as L- and K-cells, respectively.

Following RYGB, undigested nutrients bypass the proximal intestine rapidly reaching the distal small bowel leading to an increase in GLP-1 levels by nutrient stimulation of L-cells [15]. GLP-1 stimulates insulin secretion from the pancreas, increases insulin sensitivity, and inhibits glucagon, thus reducing gluconeogenesis and hepatic glucose output. GLP-1 is also an anorexigenic hormone that acts centrally to increase satiety and reduce appetite [16]. It is a combination of these responses from increased levels of GLP-1, which is thought to play a pivotal role in the metabolic changes seen after RYGB.

GIP is primarily secreted in the proximal intestine, as this is where the majority K-cells are located and have various physiological effects including both postprandial secretion of insulin and glucagon release during hypoglycaemia or a euglycaemic state [17]. Following RYGB, the role of GIP is more unclear as there are conflicting reports of GIP levels with some studies showing an increase whilst others show decreased levels or no change at all [15, 18, 19]. Nevertheless, it is hypothesised that the blunting in levels of GIP observed in some studies following RYGB contributes to the antidiabetogenic effects of this type of surgery.

Various studies have investigated the changes in gut hormones following EndoBarrier implantation including a study by de Jonge et al. of 17 obese patients with T2DM who received the device for 6 months [20]. Fasting and postprandial levels of glucose, glucagon, and insulin were recorded as well as postprandial levels of GLP-1 and GIP. The authors found a reduction in both fasting and postprandial levels of glucose following EndoBarrier implantation, which coincided with a reduction in glucagon levels, although insulin levels were unchanged. Postprandial levels of GLP-1 increased significantly and conversely; GIP levels were found to decrease at 6 months. These findings are similar to the changes in gut hormones post-RYGB previously described.

In contrast to these findings, a small study by Koehestanie et al., in which fasting GIP, GLP-1, and ghrelin levels were measured at baseline, 1 week and 4 weeks in 12 obese diabetic patients, found no significant changes in GIP. Moreover, levels of GLP-1 appeared to decrease 1-week postimplant followed by an elevation back to baseline levels in the following 3 weeks [21]. There was no correlation identified between gut hormone changes and reductions in body weight and BMI.

Rohde et al. compared the effect of the EndoBarrier on postprandial physiology in 10 obese patients with normal glucose tolerance (NGT) and 9 age-, body weight-, and BMI-matched patients with T2DM. Parameters investigated included insulin, glucose, glucagon, gut hormone secretion, gall bladder emptying, and appetite and food intake using liquid mixed meal test and a subsequent ad libitum meal test at baseline, 1 week and 26 weeks following EndoBarrier implantation [22]. Basal plasma concentrations of GLP-1, GIP, and PYY were similar in the two groups before EndoBarrier implantation, and the device did not appear to affect basal concentrations significantly in any of the groups. Small but significant increases were observed in postprandial levels of GLP-1 and PYY levels at weeks 1 and 26 in the patient group with T2DM but not in those with NGT, and overall, the EndoBarrier did not appear to have any impact on levels of insulin, glucose, or glucagon following implantation.

Levels of ghrelin appear to increase following the EndoBarrier device in clinical trials, which contradict findings post-RYGB where a reduction in ghrelin levels is often seen [21, 23]. A potential explantation for this is that ghrelin is predominantly released from P1/D1 cells in the upper stomach and fundus, a part of the GI tract which is unaffected by the EndoBarrier implant unlike in RYGB. The rise in ghrelin could be a physiological response to dieting or in response to weight loss induced by the EndoBarrier. Clearly, larger numbers are required in order to draw any firm conclusion on the effects of EndoBarrier on the gut hormones.

### 4.2. Bile Flow Modulation

Bile acids (BAs) are believed to play an integral role in regulating satiety as well as influencing lipid, cholesterol, and glucose metabolism through complex interactions, which include stimulating the secretion of incretin hormones, GLP-1 and PYY, and disruption of the gut microbiota [24]. RYGB surgery appears to impact BA homeostasis by altering the enterohepatic circulation leading to increased levels of plasma BAs postsurgery compared with at baseline [25]. Potentially, this is another mechanism to explain the higher levels of incretin hormones seen in postbariatric surgery.

Fibroblast growth factor-19 (FGF-19) is a potent stimulator of BA synthesis, and in a small study of 30 obese patients with T2DM, levels were found to be markedly increased following EndoBarrier implantation for 10 months in these individuals [26]. This might provide a partial mechanism on how the device elicits its effects on improvements in glycaemic control.

Free BAs also interact closely with the microbiota found in the small intestine, so increased concentrations of these BAs may not only influence the overall number of bacteria in this region but also their composition.

### 4.3. Gut Microbiota

The gut microbiome has been implicated in numerous disease processes, and obesity is no different. Manipulation of the host gut microbiome using faecal transplantation has been shown to alter host phenotype as evidenced by improvements in insulin resistance observed in obese individuals following transplantation with lean microbiota [27]. Obese individuals have significant differences in their gut microbiota compared with lean individuals. Two particular groups of bacteria, which have found to be both beneficial and dominant in the gut, are the Bacteroidetes and the Firmicutes. A reduction in the proportion of Bacteroidetes has been observed in obese individuals when compared with lean individuals, and these species of bacteria appear to increase postbariatric surgery [28, 29]. Increased levels of Actinobacteria have also been found in observational studies of the gut microbiome of obese individuals compared with their lean twin [30].

Following RYGB, the gut microbiota alters with an increase in bacterial richness as a consequence of the changes in pH levels in the proximal small bowel, alteration in gastric motility and nutrient flow [31].

Numerous studies have shown an increase in certain bacterial species, which include Gammaproteobacteria following RYGB [32, 33]. Further explorations into the changes in gut microbiome following surgery are required, and this might in turn uncover new therapeutic strategies for the treatment of obesity and diabetes. To date, there have been no studies looking at the impact of the EndoBarrier on the gut microbiota, but one might hypothesize that similar microbial changes may be seen as in post-RYGB surgery.

### 4.4. Gastric Emptying

The EndoBarrier's effect on gastric emptying was investigated in a small study of 25 patients [34]. A delay in gastric emptying at 16-week post-EndoBarrier implant was found when compared to baseline, but this did not appear to correlate with any clinical outcomes such as weight loss or glycaemic control.

## 5. Safety Profile

By far, the most commonly reported side effect of the device is GI upset, including abdominal pain and nausea. These symptoms usually resolve as the patient acclimatises to having the device in situ, but a minority of patients (2%) are unable to tolerate these symptoms leading to early device removal. The other complications include GI bleeding (1.5%) and device migration (1.4%). Rarer complications such as cholestasis and pancreatitis can also occur.

Betzel et al. published safety data on 185 patients from 2011 to 2014 who received the EndoBarrier for one year [35]. In 31% of cases, devices were removed prematurely and predominantly from abdominal discomfort and nausea, with more serious adverse events reported as gastrointestinal bleeding, device migration, obstruction, and the development of liver abscesses.

Liver abscesses pose the most serious complication associated with the EndoBarrier with most cases reported late during the course of treatment, that is, towards the time of explanation at 9–12 months. The German DJBL registry reported 1 case in 66 patients who had received the EndoBarrier for one year having previously reported 4 cases in a 235 patient registry [36]. Three were documented at explantation with the other one occurring following early removal for device dislocation. All were managed with antibiotics and/or drained with no permanent sequelae.

The ENDO Trial was a multicenter, double-blinded, and randomised trial in the US to evaluate the safety and efficacy of the EndoBarrier on glycaemic control. Unfortunately, in March 2015, the Food and Drug Administration (FDA) halted the trial due to the development of 7 liver abscesses (3.5%), which was much higher than anticipated. The cause for these liver abscesses is unclear, but the theory is that the EndoBarrier creates a nidus of infection, which may spread to the liver bed.

Postmarket surveillance data from GID reports an incidence of 1%, which is also supported by the data from a worldwide registry established in 2017 by the Association of British Clinical Diabetologists [37]. From 492 EndoBarrier patients, there were 6 reported cases of liver abscesses. Early removal of device because of GI bleeding was 4%. Device migration was 3% with liner obstruction rare only accounting for 0.3% of cases.

## 6. Future Developments

Currently, the EndoBarrier is licensed for 1 year after which point it should be removed. A certain issue which arises is that the vast majority of patients may then lose the beneficial effects on glycaemic control and weight loss, which the device may have been exerting whilst in situ resulting in worsening of their diabetes and an increase in their BMI. An Australian study found that in 30 patients who were followed up for 6-month period immediate postremoval of the EndoBarrier, 72% gained weight with only 5 patients maintaining their weight loss and 4 patients losing further weight [38]. In the same study, 51 patients were followed up for a period of >6 months following explant with 69% regaining their weight and only 5 patients maintaining their weight and with 7 patients losing further weight. The study did not comment on how these particular patients managed to maintain their weight loss or indeed lose further weight.

To prevent this regression, occurring future therapeutic strategies may entail reimplantation of the device or developing a prototype that can remain in situ for longer than 1 year. GID has previously reported data demonstrating the feasibility and safety of reimplantation of the EndoBarrier in 5 patients who initially completed 12 months of EndoBarrier treatment but then proceeded to have the device reimplanted after 4 months for another 12 months. HbA1c fell from a baseline of 9.1% to 6.7% after the 1st explantation and from 7.8% upon 2nd implantation to 7.1% at explant with no reported complications [39]. Clearly, the numbers in this study are very small, but reimplantation of the EndoBarrier might be another treatment option to maintain the effect of the device.

A second-generation EndoBarrier device with a 1 mm increase in barb length was trialed in 80 patients in Chile. The subjects initially consented to the implant for one year but were then given the opportunity to keep the device in for up to 3 years if tolerated [40]. The percentage excess weight losses in the complete population at 52 weeks (71 patients), 104 weeks (40 patients), and 156 weeks (11 patients) were 44 ± 16, 40 ± 22, and 39 ± 20, respectively (*p* < 0.001). There were 17 T2DM subjects enrolled in the study with baseline HbA1c of 7.1 ± 1.6%, which significantly decreased to 6 ± 0.9 and 5.7 ± 0.7% after 12 and 24 months, respectively. Two diabetic subjects managed to complete 36 months of follow-up, and both maintained an HbA1c below 6%.

An ideal situation is the creation of a device which could remain in situ for longer thus providing a more permanent solution for these patients, but for this to happen, one must consider how to combat the unwanted side effects associated with having a duodenal sleeve implanted long term.

## 7. Conclusion

The EndoBarrier appears to be a promising device for the management of obese patients with type 2 diabetes, which is designed to mimic the clinical and physiological effects of bariatric surgery. It can induce moderate weight loss and improvements in glycaemic control in patients with an acceptable safety profile. However, currently, there is a paucity of data available with the majority of trials published containing small numbers of patients or from an unrandomised setting.

Its current position in the treatment algorithm for obesity and diabetes is unclear, and larger randomised controlled trials are required to help to try to answer this question. At present, we see the device's utility in the following situations:

- In obese patients with poorly controlled T2DM who decline or are unfit for surgery
- An adjunct to surgery to induce weight loss in the superobese prior to bariatric surgery
- To facilitate glycaemic improvement in patients requiring elective surgery but whose poor diabetes control precludes this surgical procedure from being performed

The EndoBarrier in its current form is unlikely to replace bariatric surgery but may act as a complementary intervention within the arsenal of antidiabetes/antiobesity therapies. Future work in this field should focus on developing the device so that it offers longer treatment duration whilst also improving its safety profile and tolerability. Such a minimally invasive device may then carry the potential to address some of the broader needs of the global diabesity population.

## Figures and Tables

**Table 1:** Advantages and disadvantages of the EndoBarrier procedure.

Advantages	Disadvantages
Can be performed without the need for a general anaesthetic under conscious sedation	Two operators required for both implant and explant.
Easy to perform	Fluoroscopy is essential, and this involves a small dose of radiation to the patient.
Patient can be discharged at home the same day	Patients must take protein pump inhibitors for the duration of treatment with the device.
Easily reversible	Patients must commence a liquid diet prior to EndoBarrier placement and for a short duration afterwards.

**Table 2:** Summary of RCTs on the EndoBarrier.

Study	Number of patients	BMI	Implant duration (weeks)	Weight loss	Change in HBA1c	Stent removal rate
Gersin et al. [6]	47; 21 in treatment arm	46 kg/m^2^	12	−8.2 kg ± 1.3 kg in treatment arm versus 2 kg ± 1.1 kg in sham arm	Not an endpoint	38%
Koehestanie et al. [4]	73; 34 in treatment arm	35 kg/m^2^ device; 37 kg/m^2^ in control	26	10.6 kg device; 5.3 kg in control	−1.3% versus 0.4% in control	3%
Rodriguez-Grunert et al. [7]	18; 12 in treatment arm	39 kg/m^2^	24	−10.2 kg ± 1.3 kg in device arm versus 7.1 ± 4.3 kg in sham arm	−2.4 ± 0.7% versus −0.8 ± 0.4% in control	25%
Schouten et al. [5]	41; 30 in treatment arm	49 kg/m^2^	12	19% in device; 6.9% in control	−1.1% versus 0.4% in control	15%
Tarnoff et al. [8]	35; 29 in treatment arm	42 kg/m^2^ in device; 40 kg/m^2^ in control	12	−10.3 kg ± 3.2 kg versus 2.6 kg ± 3.5 kg in the control group	Not an endpoint	20%
